# Phosphorylation of the Twist1-Family Basic Helix-Loop-Helix Transcription Factors Is Involved in Pathological Cardiac Remodeling

**DOI:** 10.1371/journal.pone.0019251

**Published:** 2011-04-29

**Authors:** Shuangshuang Lu, Junwei Nie, Qing Luan, Qiuting Feng, Qi Xiao, Zai Chang, Congjia Shan, Daniel Hess, Brian A. Hemmings, Zhongzhou Yang

**Affiliations:** 1 The Ministry of Education Key Laboratory of Model Animal for Disease Study, Model Animal Research Center, Nanjing University, Nanjing, China; 2 The Friedrich Miescher Institute for Biomedical Research, Basel, Switzerland; Institute of Zoology, Chinese Academy of Sciences, China

## Abstract

**Background:**

The Twist1-family basic helix-loop-helix (bHLH) transcription factors including Twist1, Hand1 and Hand2, play an essential role in heart development and are implicated in pathological heart remodeling. Previously, it was reported that these bHLH transcription factors can be regulated by phosphorylation within the basic-helix I domain, which is involved in developmental processes such as limb formation and trophoblast differentiation. However, how phosphorylation of Twist1 family functions in post-natal heart is elusive.

**Principal Findings:**

Here, we generated transgenic mice with over-expression of Hand1 and Twist1 mutants (to mimic or to abolish phosphorylation) in cardiomyocytes and found pathological cardiac remodeling leading to heart failure and sudden death. Gene expression profile analysis revealed up-regulation of growth-promoting genes and down-regulation of metabolic genes. It is well known that aberrant activation of Akt signaling causes pathological cardiac remodeling and results in heart failure. The basic-helix I domain of Twist1 family members contain Akt substrate consensus motif and may be downstream targets of Akt signaling. Using biochemical analysis, we demonstrated that Hand1 and Twist1 were phosphorylated by Akt in the basic-helix I domain. Phosphorylation of Hand1 regulated its transcriptional activation of luciferase reporter genes and DNA binding ability.

**Conclusions:**

This study provides novel insights into the regulation of Twist1 family in cardiac remodeling and suggests that the Twist1 family can be regulated by Akt signaling.

## Introduction

The Twist family of basic helix-loop-helix (bHLH) transcription factors including Twist1/2, Hand1/2, Scleraxis and Paraxis, play a variety of roles in both embryonic development and diseases [1], [2], [3], [4]. Among the Twist1 family members, Twist1 and Hand1 (also termed eHand, Hxt and Thing1) have been intensively studied [2], [5], [6], [7], [8]. Biochemical studies have demonstrated that Twist1 and Hand1/2 formed hetero-dimers or homo-dimer with E12, E47 and between themselves to activate or suppress transcription of downstream target genes through binding E-box sequences such as the degenerate Thing1-box, CGTCTG, in the *cis*-regulatory elements of target genes [1], [6], [9], [10], [11].

Protein phosphorylation plays an important role in regulating the activity and function of Twist1 family members. Firulli and colleagues first reported that PKA, PKC and PP2A (containing the B56δ regulatory subunit) could regulate phosphorylation of Hand1 and Hand2, which controled trophoblast cell line RCHO1 differentiation [12]. In a following study, Firulli's group demonstrated that phosphorylation regulates Twist1-Hand1 dimerization and the results suggested that the partner choice of Twist1 family protein dimerization is controlled by post-translational modification, which is crucial for normal development [9]. Hand1 was also reported to be phosphorylated by polo-like kinase Plk4 (Sak), which regulated Hand1 cellular localization and determined cell fate [13]. A recent study showed that phosphorylation of Twist1 affected its dimer affinity for a given partner and modulated the DNA binding affinity, which might control limb development [10].

Both Hand1 and Hand2 are essential for heart development as either germ-line or cardiac-specific deletion of Hand1 and Hand2 in mice causes heart defects and mortality [5], [8], [14], [15]. Hand1 was also found to play a role in pathological heart remodeling in human and rodent models [16], [17]. Although phosphorylation of Twist1 family is important for embryonic development, its role in post-natal heart function and remodeling remains elusive. For this purpose, we generated transgenic mice with over-expression of Hand1 and Twist1 wild-type and mutants (to mimic and to abolish phosphorylation) in cardiomyocytes and found pathological heart remodeling.

Akt signaling plays an important role in heart function, which has been intensively investigated in the past decade [18], [19], [20], [21], [22], [23]. Aberrant activation of Akt signaling gives rise to pathological cardiac remodeling including hypertrophy and heart enlargement that leads to heart failure [24], [25], [26], [27], [28], [29]. However, the downstream targets of Akt signaling involved in heart remodeling are not well known [30].

The basic helix I domain of Twist1 family proteins contain a well-conserved Akt substrate consensus motif suggesting that they are putative Akt substrates. Through biochemical and cell transfection assays, we demonstrated that both Twist1 and Hand1 could be phosphorylated by Akt, and phosphorylation regulated their transcriptional activity and DNA binding affinity. This study provides novel insights into the regulation of Twist1 family in cardiac remodeling and suggests that the Twist1 family can be regulated by Akt signaling.

## Results

### Mice over-expressing Hand1 and Twist1 mutants in cardiomyocytes developed pathological cardiac remodeling

**Figure 1:**
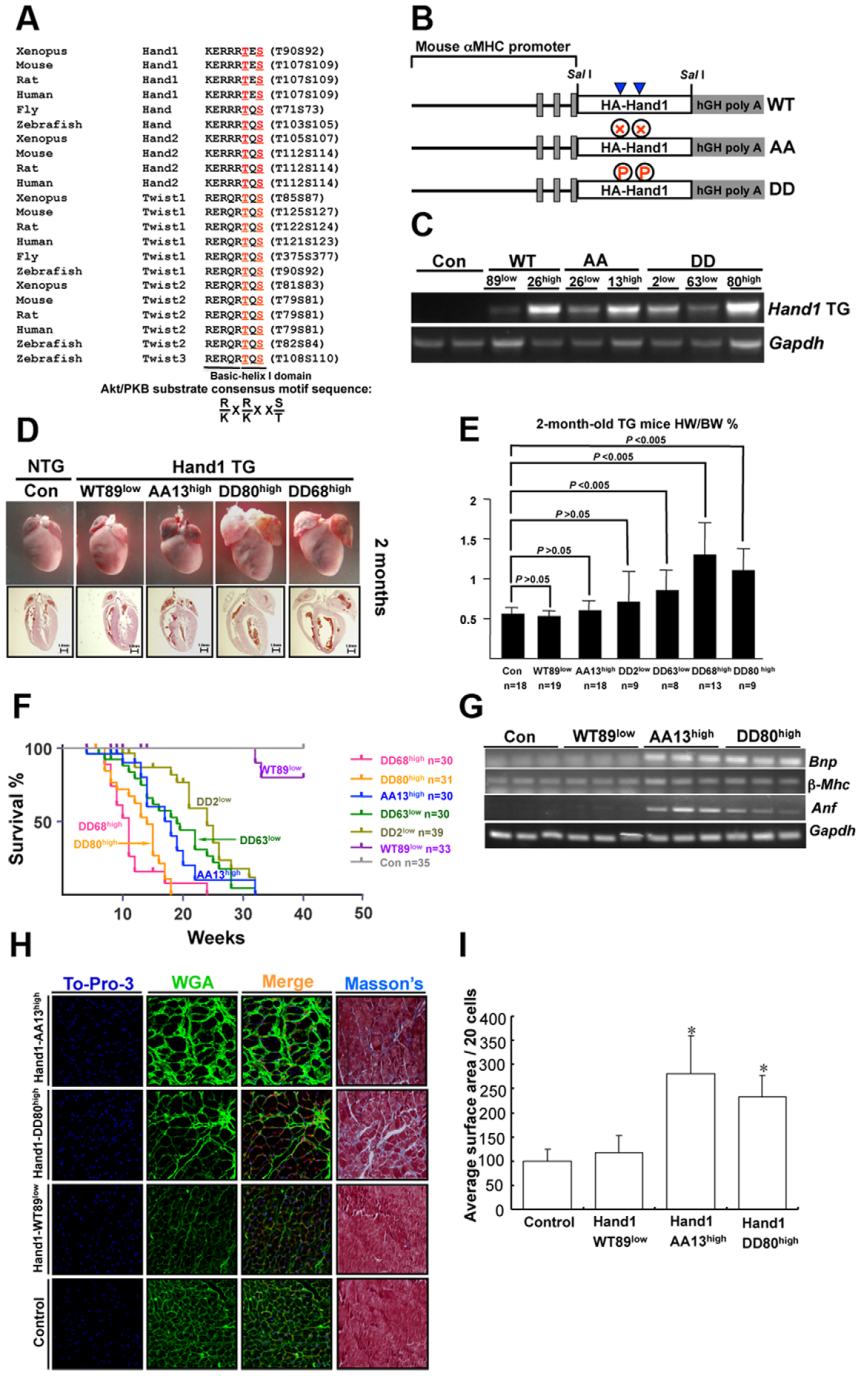
Generation and characterization of Hand1 TG mice. **A**. Alignment of the basic helix I domain among Twist1 family proteins in different species. This motif (KERRRTES or KERRRTQS) is well conserved in the Hand/Twist proteins in fly, zebrafish, xenopus, mouse, rat and human. Akt/PKB substrate consensus motif is K/R X K/R X X T/S (T and S are residues for phosphorylation) and can be identified in this motif. Letters in red (T or S) indicate phosphorylation sites that are located in the basic-helix I domain. In both fly and zebrafish, there is only one Hand protein. **B**. Schematic representation of the three constructs for generation of Hand1 TG mice. Solid blue tri-angles indicate two phosphorylation sites. Abbriviations: WT, wild-type; AA, Hand1-T107A;S109A mutant; DD, Hand1-T107D;S109D mutant; TG, transgenic. **C**. Expression levels of Hand1 in TG hearts detected with RT-PCR. *Gapdh* was used as control. DD^high^ indicated lines with high *Hand1* expression while DD^low^ showed lines with low *Hand1* expression. **D**. Gross anatomy of Hand1 TG heart at 2 months. Note the hypertrophy and dilation phenotype of Hand1-DD^high^ hearts. **E**. Heart weight/body weight ratio of Hand1 TG mice at two months. **F**. Survival curve of Hand1 TG mice. **G**. Expression of *Bnp*, *β-Mhc* and *Anf* in Hand1 TG hearts at 1 month. *Gapdh* is for control. **H**. Histological analysis of left ventricular free wall of Hand1 TG hearts. To-Pro-3 stained nucleus; WGA staining displayed the size of cardiomyocytes; Masson's staining revealed fibrosis. Note the cardiomyocyte hypertrophy and fibrosis in Hand1-AA and –DD hearts. **I**. Measurement of cardiomyocyte surface area of TG mice. * *P*<0.05 v.s. control.

The Twist1 family members of Hand1, Hand2, Twist1 and Twist2 possess a well-conserved basic-helix I motif that contains threonin and serine (Figure 1A). Therefore, phosphorylation of Twist1 family may play a role in post-natal heart function. To investigate this, we first generated transgenic (TG) mice expressing Hand1-WT, -AA and -DD specifically in cardiomyocytes (Figure 1B). Several lines of TG mice were obtained with distinct expression levels of all three Hand1 genes (Figure 1C and Figure S1C). Two mice lines expressing high-level Hand1-DD (designated DD80^high^ and DD68^high^) displayed heart hypertrophy at 1 month that became more severe at 2 months (Figure 1D–F). The majority of these mice died at 2–3 months and showed heart hypertrophy and dilation (Figure 1D–F). Two lines with a relatively low Hand1-DD expression (designated DD2^low^ and DD63^low^) developed heart hypertrophy at 2 months and the majority of these mice died at 4–5 months (Figure 1E and 1F). Echocardiography (Echo) tests on Hand1-DD mice indicated a reduction in heart contractility and function at 8–10 weeks (Table 1). These results showed a dose-dependent effect of Hand1-DD. Two TG lines of Hand1-AA (including Hand1-AA13^high^ and –AA26^low^) displayed similar phenotypes: their hearts were relatively small at 2 months, showing slight dilation and a reduction in heart function (Figure 1D–F and Table 1). At 4–5 months, the Hand1-AA TG mice developed heart dilation and enlargement. Hand1-AA and –DD TG mice showed increased expression of *Bnp*, *βMhc* and *Anf* at 1 month indicating pathological heart remodeling (Figure 1G). TG mice with over-expression of Hand1-WT in heart did not display apparent phenotype by 32 weeks, which was in consistence with a recent report showing that Hand1-WT TG mice displayed mild hypertrophy but were predisposed to cardiac arrhythmia [31]. Immunofluorescence microscopy and Masson's staining on frozen sections of control, Hand1-WT, -AA and -DD hearts displayed cardiomyocyte size and fibrosis (Figure 1H). Both Hand1-AA and -DD hearts displayed cardiomyocyte hypertrophy and fibrosis in the left ventricular free wall at 2 months (Figure 1H and I). These results indicate that aberrant phosphorylation (lower or higher) of Hand1 caused pathological heart remodeling.

**Table 1:** Echocardiography of 8- to 10-week-old Hand1 TG mice.

	Con(n = 5)	Hand1WT89^low^ TG (n = 5)	Hand1AA26^low^ TG (n = 5)	Hand1DD2^low^ TG (n = 6)
FS%	33.09±3.80	29.07±4.72	20.70±4.41*	17.40±8.84*
EF%	62.67±5.27	56.61±7.29	41.44±6.89	35.89±7.44*
LVID; s (mm)	2.27±0.37	2.49±0.34	2.43±0.29	3.06±0.56*
LVPW; d (mm)	0.73±0.06	0.84±0.21	0.68±0.07	0.70±0.11
LVPW; s (mm)	1.10±0.05	1.14±0.17	0.81±0.07*	0.87±0.19*
LV Vol; d (µl)	47.19±12.08	51.35±9.38	46.17±11.37	79.41±20.11
LV Vol; s (µl)	18.14±6.63	22.75±7.20	21.22±6.16	56.45±10.34

*P<0.05 compared with control.

Abbreviations: EF, ejection fraction; FS, fractional shortening; LVID, left ventricular internal; LVPW, left ventricular posterior wall; LV Vol, left ventricular volume; d, diastolic; s, systolic.

We collected hearts from these Hand1 TG mice and performed microarray analysis to study gene expression profile. We found up-regulation of several key growth-promoting genes such as *Fgf1r*, *Fgf12*, *Igf1*, *Igf1r* and *Cyclin D2* (*Ccnd2*) in Hand1-DD hearts compared to Hand1-TG hearts (Table S1). RT-PCR result confirmed increased *Cyclin D1* and *D2* in Hand1-DD hearts (Figure S1A and B). Interestingly, genes involved in oxidative phosphorylation and citrate cycle (TCA cycle) were found with reduced expression in Hand1-DD hearts (Table S1). *Cyclin D1* (*Ccnd1*) and *cyclin D2* (*Ccnd2*) were also up-regulated in Hand1-AA hearts and genes encoding collagen isoforms were high in both Hand1-DD and –AA hearts consistent with fibrosis revealed by histological study (Table S1 and Figure 1H).

**Figure 2:**
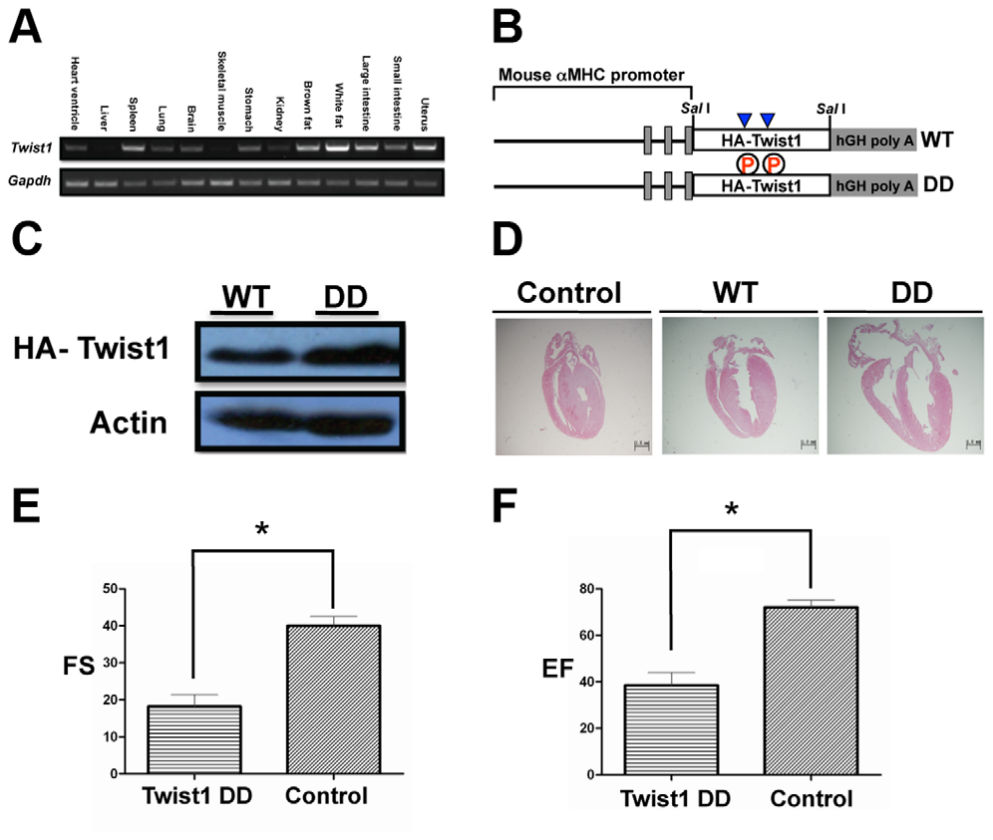
Detection of Twist1 expression, generation and characterization of Twist1 TG mice. **A**. RT-PCR detection of *Twist1* expression in mouse tissues. Note that *Twist1* is expressed in heart ventricles. **B**. Schematic representation of the two constructs for generation of Hand1 TG mice. WT, Twist1 wild-type; DD, Twist1 T125D; S127D mutant. **C**. Detection of Twist1 expression in the heart of TG mice by western analysis using HA antibody. **D**. Histological study of hearts from Twist1 TG mice (20 days). Note that Twist1-DD mice show heart hypertrophy and atrial septal defect (ASD, indicated by arrow) while WT mice have comparable heart to control (data not shown). **E** and **F**. Heart's function analysis by echocardiography.

We first detected Twist1 expression in mouse tissues by RT-PCR and the result indicated that Twist1 was ubiquitously expressed with varied amount (Figure 2A). In particular, we found that Twist1 was expressed in heart ventricles (Figure 2A). Using a similar strategy for generation of Hand1 TG mice, we obtained Twist1-WT and -DD transgenic TG mice and found that while -WT TG mice had comparable heart to control mice, Twist1-DD TG mice displayed hypertrophy and some of them had atrial septal defect (ASD) and ventricular septal defect (VSD) (Figure 2B–D). Echo analysis indicated impaired heart function in Twist1-DD mice compared to WT mice (Table 2 and Figure 2E and 2F).

**Table 2:** Echocardiography of 20-day Twist1 TG mice.

	WT (n = 6)	Twist1DD (n = 7)
IVS; d (mm)	0.59±0.02	0.72±0.03
LVID; d (mm)	2.68±0.04	2.72±0.07
LVPW; d (mm)	0.61±0.01	0.71±0.05
IVS; s (mm)	0.69±0.03	0.72±0.03
LVID; s (mm)	1.61±0.03	2.24±0.07*
LVPW; s (mm)	0.87±0.02	0.76±0.05
LV Vol; d (ìl)	26.84±0.91	28.57±1.50
LV Vol; s (ìl)	7.49±0.42	18.21±1.07**
EF %	72.02±1.25	38.43±2.11**
FS %	39.96±1.04	18.20±1.20**
LV Mass (mg)	41.31±0.72	54.98±3.46
LVMass Corrected (mg)	33.05±0.57	43.98±2.77

*P<0.05 and

**P<0.005.

Abbreviations: IVS, interventricular septum; LVID, left ventricular internal; LVPW, left ventricular posterior wall; LV Vol, left ventricular volume; EF, ejection fraction; FS, fractional shortening; d, diastolic; s, systolic.

### Hand1 and Twist1 could be phosphorylated by Akt *in vitro* and *in vivo*

In the past decade, the role of Akt signaling in post-natal heart remodeling has been intensively investigated using Akt transgenic mouse models [24], [25], [26], [27], [28], [29]. These studies have demonstrated that hyper-activation of Akt in cardiomyocytes caused severe pathological cardiac remodeling leading to heart failure [32]. Bioinformatic study using a computer program called Scansite (http://scansite.mit.edu) has predicted a well-conserved Akt substrate consensus motif in the basic-helix I domain of the Twist1 family members (Figure 1A) [33]. Gene expression analysis indicated increased expression levels of *Igf1* and its receptor *Igf1r* that activated Akt signaling pathway (Table 2). Therefore, the Twist1 family could be regulated by Akt and might be involved in heart remodeling.

**Figure 3:**
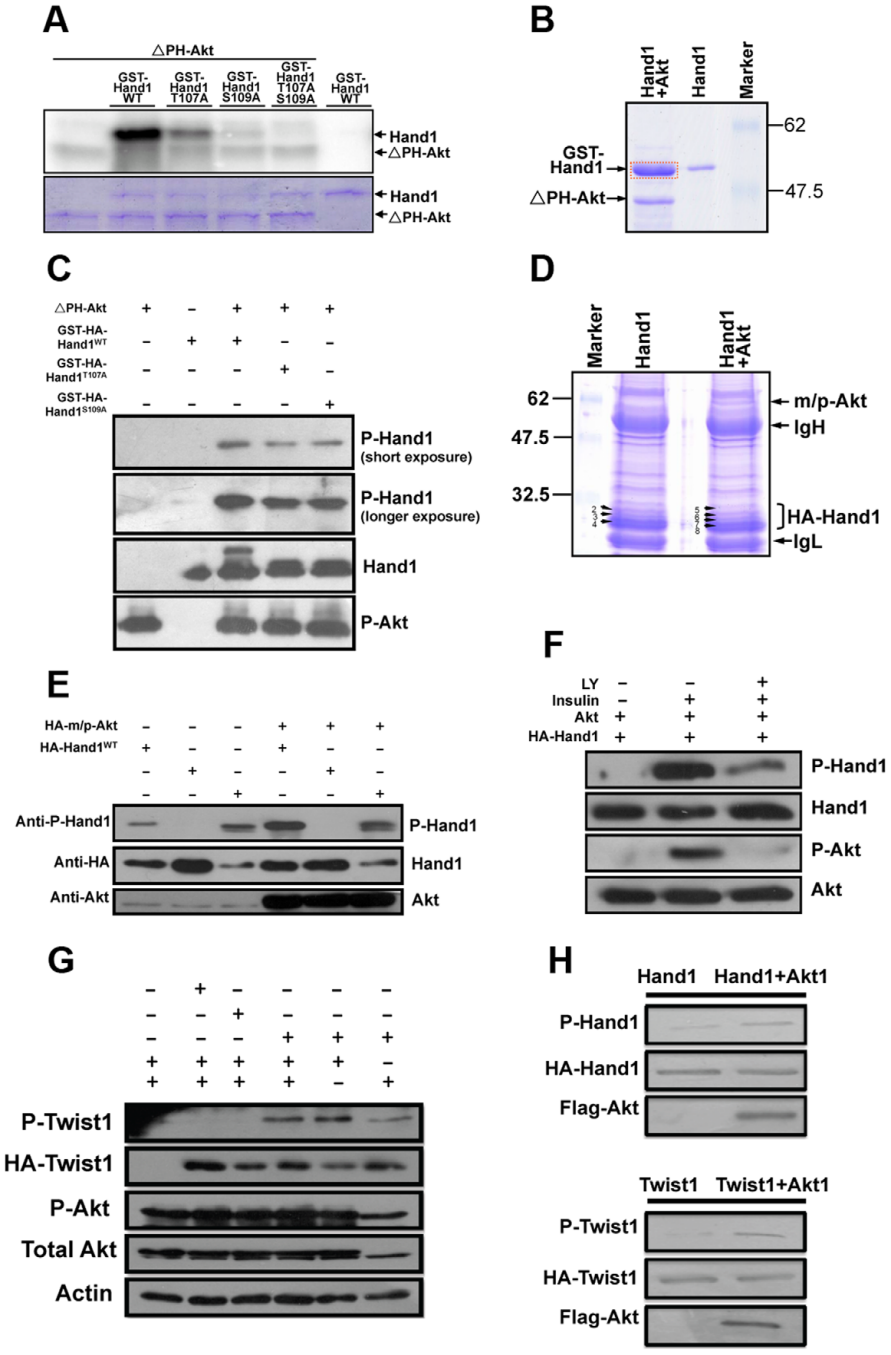
Hand1 and Twist1 could be phosphorylated by Akt *in vitro* and *in vivo*. **A**. Autoradiography of an *in vitro* phosphorylation assay showing that Akt phosphorylates Hand1 at both Thr107 (T107) and Ser109 (S109). ΔPH-Akt is an Akt mutant without the PH domain and is constitutively active. T107A and S109A are Thr107 and Ser109 mutations to alanine that block phosphorylation. **B**. SDS-PAGE gel showing separation of GST-Hand1 and ΔPH-Akt after an *in vitro* phosphorylation assay. The GST-Hand1 band (framed in red) was excised from SDS-PAGE gel and applied to Mass spectrometric analysis. The result confirmed Hand1 phosphorylation by Akt. **C**. Western blot using phospho-Hand1-specific antibody to detect Hand1 phosphorylation by Akt. In the GST-HA-HandT107A protein, S109 could be phosphorylated by Akt and detected with the phospho-specific antibody. The similar effects were observed in GST-HA-Hand1S109A protein. **D**. Mass spectrometric analysis of Hand1 phosphorylation by Akt in transfected HEK293 cells. HA-Hand1 proteins were pulled down by immunoprecipitation (IP) and were separated with SDS-PAGE gel. The bands containing Hand1 [(2]–[8)] were excised for Mass spectrometric analysis leading to identification of peptides with T107 or S109 phosphorylation in bands 5–8 (from cells transfected with HA-Hand1 together with constitutively active Akt–m/p-Akt). No phosphor-peptide was detected in bands 2–4 in the absence of Akt. IgH and Ig L mean immunoglobulin heavy and light chain. **E**. Western blotting analysis using phospho-Hand1 antibody to detect Hand1 phosphorylation in transfected cells. Akt augmented Hand1 phosphorylation. The antibody also detected Hand1-DD protein which mimics Hand1 phosphorylation but to a lesser extent while it did not recognize Hand1T107AS109A. **F**. Study of Hand1 phosphorylation in HEK293 cells with insulin to activate Akt. After transfection, cells were starved over-night by withdrawal of serum from medium and insulin was added to the cells for 15 minutes resulting in Akt phosphorylation and activation. Avtivation of Akt, in turn, phosphorylated Hand1. This is blocked by LY204002 (LY), a PI3K inhibitor. P-Akt represents Akt phosphorylation and activation. **G**. Study of Twist1 phosphorylation in HEK293 cells as for Hand1. **H**. Test of Hand1/Twist1 phosphorylation by Akt in mouse cardiomyocytes.

An *in vitro* phosphorylation assay using GST-Hand1 fusion protein demonstrated that mouse Hand1 could be phosphorylated by Akt at two residues, T107 and S109 (Figure 3A). Mutation of these two amino acids to alanine (A) in Hand1 abolished phosphorylation by Akt (Figure 3A). The phosphorylation sites were also confirmed by mass spectrometric analysis of GST-Hand1 after Akt kinase assay, which identified phopho-Hand1 peptide (Figure 3B). Western blotting analysis using a phospho-specific Hand1 antibody also showed Hand1 to be phosphorylated at both T107 and S109 (Figure 3C).

The results so far all indicated that Akt phosphorylated Hand1 *in vitro*. We next studied Hand1 phosphorylation by Akt in cultured mammalian cells by an *in vivo* biochemical assay. HA-Hand1 expressed in HEK293 cells alone or together with constitutively active Akt was pulled down by immunoprecipitation (IP) (Figure 3D). After SDS-PAGE and Coomassie blue staining, Hand1 bands were excised and analyzed by mass spectrometry (Figure 3D). No phospho-peptides were found in bands of Hand1 without co-transfection of Akt (Bands2–4 in Figure 3D), but phospho-peptides with the sequence profile of RTpESINSAFAELR and RTESpINSAFAELR (with phosphorylation on T107 or S109) were identified in bands co-expressed with Akt (Bands 5–8 in Figure 3D). Furthermore, we examined Hand1 phosphorylation by Akt with phospho-specific antibody. In cells expressing wild-type Hand1 and Hand1-DD (to mimic phosphorylation), a phospho-band could be detected and the signal became stronger in the presence of constitutively active Akt (m/p-Akt) while there was no band with cells expressing Hand1-AA (to abolish phosphorylation) (Figure 3E). In addition, we studied Hand1 phosphorylation by Akt with insulin stimulation. Cells were starved over-night by serum withdrawal, and treated with insulin to activate Akt (Figure 3F). Akt activation brought about Hand1 phosphorylation which was blocked by LY-294002 (LY), an inhibitor of PI3K-Akt signaling (Figure 3F). Similarly, Twist1 could also be phosphorylated by Akt (Figure 3G and Figure S2). Furthermore, we also tested Hand1/Twist1 phosphorylation by Akt in cultured cardiomyocytes and the results were consistent with those performed in HEK293 cells (Figure 3H). Collectively, these results indicate that Hand1 and Twist1 are *bona fide* Akt substrates.

### Akt suppressed Hand1 activation of reporter gene transcription

**Figure 4:**
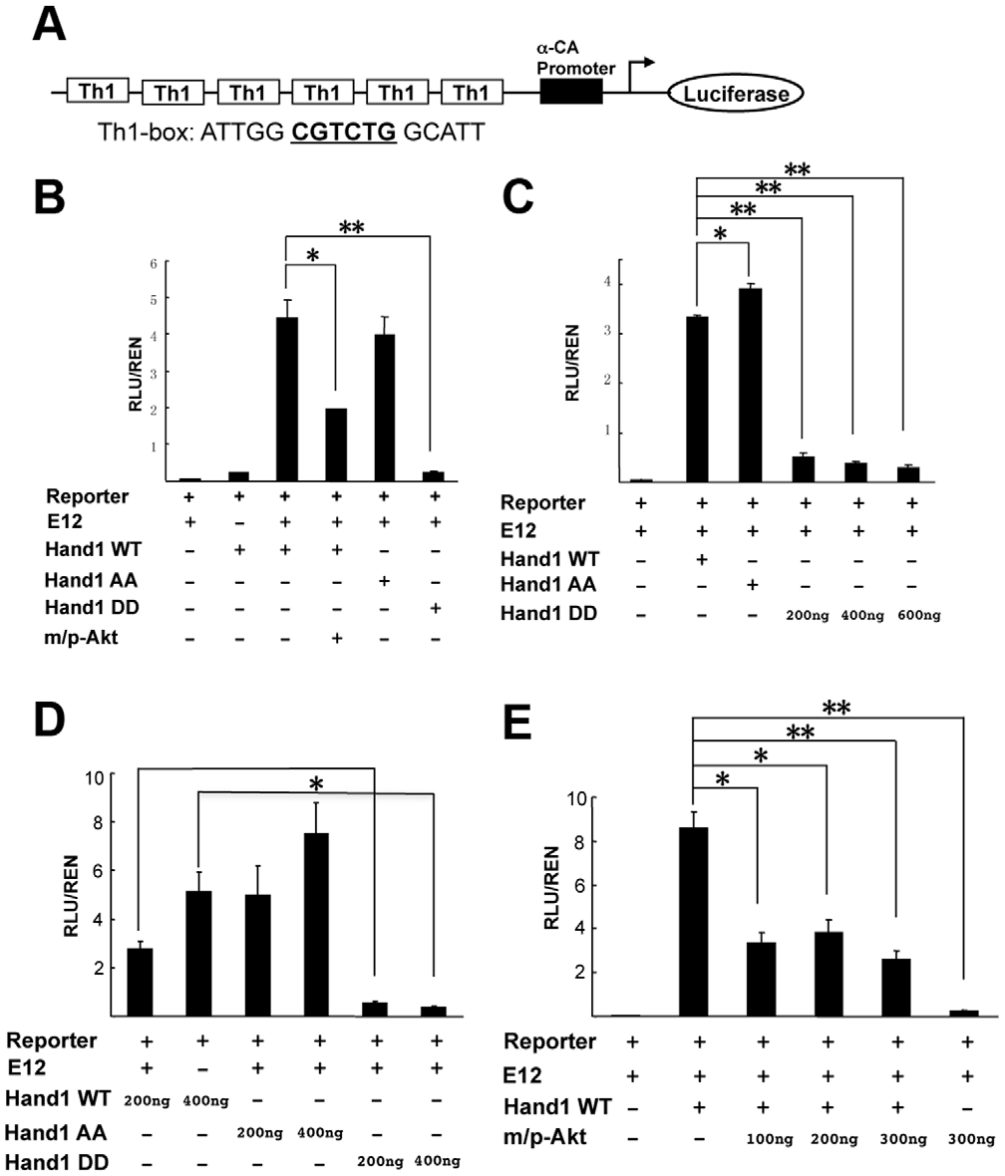
Akt phosphorylation suppressed Hand1 activation of a luciferase reporter gene. **A**. Structure of Th1-luciferase reporter. Th1 represents the Th1-box sequence (CGTCTG, D-box) that is bound by Hand1. αCA is α-cardiac actin. Six successive Th1-boxes are located upstream of αCA promoter. **B**. Hand1-WT and –AA (T107AS109A) activate the luciferase reporter gene, whilst –DD (T107DS109D) represses this activation. Akt also inhibits Hand1-WT activation of the reporter gene. **C**. Hand1-DD suppresses reporter gene activation. **D**. Effects of three Hand1 proteins on reporter gene expression. **E**. Akt action on Hand1 regulation of reporter gene expression. These assays were performed with lysates from HEK293 cells transfected with corresponding plasmids. * *P*<0.05; ** *P*<0.01.

Previous studies have shown that Hand1 forms heterodimers with class A E-factors such as E12 and E47 to activate or suppress transcription of downstream target genes via binding the “E-box” sequence (CANNTG) or the degenerate “D-box” sequence (CGTCTG) [2], [6], [7], [11]. Hand1 and Hand2 are close isoforms, and their bHLH motives are nearly identical. It has been found that the basic domain of Hand2 (RRR109-111) was crucial for DNA binding and mutation of RRR109-111 to EDE abolished Hand2 DNA binding and activation of luciferase reporter gene transcription [34]. Akt1 phosphorylated Hand1 in the basic-helix motif and might have similar function as Hand2 RRR109-111EDD mutant on reporter gene expression. To investigate this, we performed a luciferase reporter assay with an artificial Th1-luciferase reporter plasmid containing six successive Th1(Hand1) binding boxes (CGTCTG, D-box) followed by a basic *α-cardiac actin* (*α*-CA) promoter and luciferase cDNA [6], [11], [13] (Figure 4A). As shown in Figure 4B, E12 and Hand1 alone caused very weak activation of luciferase expression whilst their combination strongly activated the luciferase gene. Akt suppressed Hand1/E12 activation of luciferase gene expression (Figure 4B, E). Consistently, the phospho-mimic form of Hand1, Hand1-DD, robustly suppressed luciferase gene activation (Figure 4B–D). The non-phospho-Hand1, Hand1-AA, showed an equal or even higher ability to activate luciferase expression that was dose-dependent (Figure 4B–D).

### Phosphorylation reduced Hand1 DNA binding ability

**Figure 5:**
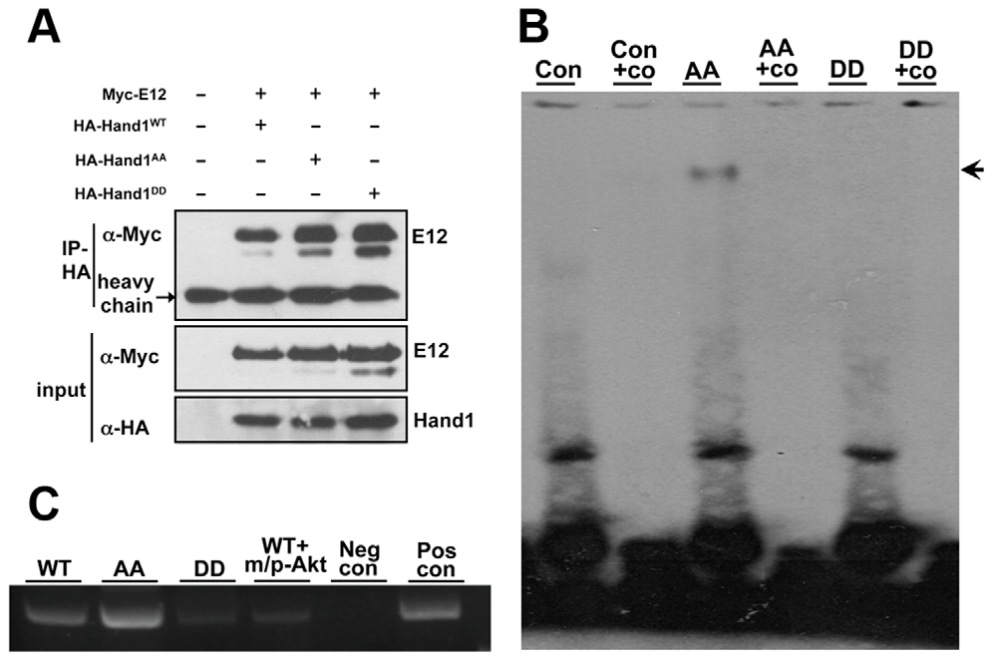
Hand1 phosphorylation did not affect heterodimer formation with E12 but abolishes DNA binding. **A**. HA-Hand1-WT, -AA and -DD plasmids were co-transfected with Myc-E12. HA-Hand1 was pulled down by HA-IP and blotted with an antibody against Myc. Myc-E12 was found to bind to all three HA-Hand1 proteins. **B**. Gel shift assay. Hand1-AA showed strong DNA binding while phosphorylation (Hand1-DD) abolished the DNA binding (indicated by arrows). Co represents competition oligo. **C**. ChIP assay. Hand1 wild-type and mutants was expressed with or without Akt in HEK293 cells. Using nuclear extracts from these cells, ChIP assay was performed and the results showed that Hand1-AA had stronger DNA binding than Hand1-DD. Akt also reduced Hand1 DNA binding ability. Abbreviations: Neg con, negative control; Pos con, positive control.

Using immunoprecipitation (IP) assay, we found that phosphorylation of Hand1 did not alter its ability to bind E12 (Figure 5A). Next, we studied Hand1/E12 DNA binding ability after phosphorylation by gel shift and ChIP assay. As shown in Figure 5B, Hand1-DD was abolished from DNA binding while Hand1-AA displayed strong DNA binding ability. Furthermore, ChIP assay also demonstrated that Hand1-DD had a much weaker DNA binding ability than Hand1-WT and -AA (Figure 5C). Consistently, Akt reduced Hand1 DNA binding ability (Figure 5C). Similar to reporter gene regulation assay, phospho-Hand1 showed the same effects on DNA binding as Hand2RRR109-111EDE mutant [34].

## Discussion

Our study has demonstrated that phosphorylation of Twist1 family played a critical role in heart remodeling in mouse models and provided insights into the regulation of Twist1 family in heart remodeling. This study indicated that the Twist1 family was involved not only in embryonic heart development but also in post-natal heart function. Previous studies have revealed an essential role of Twist1 for embryonic development including cardiac neural crest and valve morphogenesis [35], [36], [37]. Our work suggests that phosphorylation of Twist1 family may also regulate embryonic development. Work performed by Olson's group supports this hypothesis. They have shown that the basic domain of Hand2 (RRR109-111) is crucial for DNA binding and regulation of target gene expression. Mutation of RRR109-111 to EDE abolished Hand2 DNA binding and activation of luciferase reporter gene transcription [34]. In mice, RRR109-111EDE mutation caused embryonic lethality at around E10.5 and right ventricular hypoplasia [38]. Obviously, mutation of the basic domain disrupts the consensus motif of Hand2 and abolishes phosphorylation. The Twist1 family members of Hand1, Hand2, Twist1 and Twist2 possess a well-conserved basic-helix I motif that contains threonin and serine. Among them, Hand1, Hand2 and Twist1 were previously reported to be phosphorylated by PKA, PKC and Plk4 at the two residues of threonin and serine [9], [12], [13]. Another study showed that Hand2 could be phosphorylated at the same residues by Akt [39]. Phosphorylation of these Twist1 family members regulates developmental process and cell fate determination such as limb formation and trophoblast differentiation [9], [10], [12], [13]. Studies of mutant mice have shown that Hand1, Hand2 and Twist1 play a critical role in heart development [5], [8], [14], [15]. Hand1 and Hand2 were found involved in the development of cardiomyopathy in rodents and human [16], [17]. In the future, generation of inducible/conditional knock-in mutation mice will make it possible to explore the role of Twist1 family phosphorylation in post-natal heart function.

In this study, we found that phosphorylation of Hand1 suppressed its transcriptional activity. Previously, Firulli and colleagues performed comprehensive study of Twist1-AA and Twist1-DD mutation on transcriptional activity regulation and their results indicated that both reduced gene transcription of luciferase reporters [10]. In human patients with cardiomyopathy as well as rodent models of cardiac hypertrophy, Hand1 gene expression levels were found reduced suggesting a correlation between Hand1 levels and heart remodeling [16], [17]. Combined with our transgenic mouse models, these studies suggest that Twist1 and Hand1 may play a growth-inhibitory role in post-natal heart and phosphorylation may release their inhibitory effects leading to hypertrophy. Data obtained from microarray analysis supports this hypothesis because we found increased expression levels of growth-promoting genes including *Fgf1r*, *Fgf12*, *Igf1*, *Igf1r*, *Cyclin D2* in Hand1-DD heart. Interestingly, the genes involved in oxidative phosphorylation and TCA (citrate) cycle were found with reduced levels in Hand1-DD heart suggesting that decreased metabolism may result in heart failure [40].

Previously, it was reported that Akt phosphorylated Hand2 *in vitro* and phosphorylation negatively regulated Hand2 DNA binding and activation of luciferase reporter gene expression [39]. Our results are consistent with this study. However, how Hand2 phosporylation functions in heart development and remodeling remains unknown. In the future, it will be necessary to study this Akt-Hand2 relationship in mice because of distinct expression pattern of Hand1 and Hand2 in left and right ventricles [2].

The role of Akt in pathological heart remodeling has been intensively investigated by studying transgenic mice with Akt over-expression in cardiomyocytes [18], [20], [22], [23], [24], [25], [26], [27], [28], [29], [41], [42], [43], [44]. These studies have demonstrated that aberrant activation of Akt in cadiomyocytes gave rise to pathological hypertrophy. On the other hand, reduction of Akt activity in cardiomyocytes resulting from deletion of its upstream kinase, PDK1, also caused pathological cardiac remodeling (dilated cardiomyopathy and heart enlargement) [45], [46]. Using *αMhc*-Cre, we deleted PDK1 in cardiomyocytes and obtained similar results (data not shown). Gene expression study indicated increased expression levels of *Igf1* and its receptor in Hand1-DD heart, which suggested that IGF1-PDK1-Akt-Hand1 positive feedback loop might contribute greatly to the phenotype. This needs to be tested in the future. Taken together, these studies suggest that the Twist1 family might be the key downstream players in heart hypertrophy/dilation induced by abnormal Akt signaling (enhancement or reduction).

Intriguingly, Hand1S109G mutation in the phosphorylation motif has been identified in human VSD heart sample pinpointing the role of phosphorylation regulation of Hand1 in heart development [47]. In the future, it will be worthwhile to search for Hand1 mutations in patients with pathological heart remodeling, such as hypertrophic and dilated cardiomyopathy (HCM and DCM).

In summary, we have identified a novel regulatory mechanism of Twist1 family involved in cardiac remodeling. Our work suggests that modulation of Akt activity (enhancement or reduction) by agonists or inhibitors may have therapeutic application on pathological cardiac remodeling. Meanwhile, this study puts caution on pharmacological development of inhibitors to suppress PI3K-Akt signaling against tumor growth in that reduction of Akt signaling may have severe cardiac toxicity.

## Materials and Methods

### Chemicals and antibodies

LY-294002 (cat# 440202) was from Calbiochem and insulin from Sigma. The following antibodies were from Cell Signaling Technology (CST): total Akt (9272), pSer473 phospho-Akt antibody (9271), Twist1 (4119) and Tubulin (2148). Pan-actin antibody (MS-1295-P0) and HRP-linked secondary antibodies (Prod #31460 and Prod #31430) were from Thermos Scientific. The HA (FMI 42F13) and Myc (9E10) antibodies were from a hybridoma cell culture supernatant maintained in the authors' laboratory.

### Plasmids construction

Mouse *Hand1* cDNA was cloned from E14.5 placenta by RT-PCR and the two primer sequences were: forward, *BamH*I **GGA TCC** ACC (*HA tag*) *ATG GCT TAC CCA TAC GAT GTT CCA GAT TAC GCT TCG* (Hand1-5′) AAC CTC GTG GGC AGC TAC GCA; reverse, *BamH*I **GGA TCC** (Hand1-3′) TCA CTG GTT TAG CTC CAG CGC. The PCR product is 699 bp encoding a protein of 227 amino acids (including12 amino acids of the HA tag). The PCR product was further cloned into pGEMT-easy to generate pGEMT-easy-HA-mHand1 and verified by sequencing. For mammalian expression vector construction, pGEMT-easy-HA- mHand1 was digested with *BamH*I and the fragment (699 bp) released was subcloned into pCMV5 at a *BamH*I site, resulting in pCMV5-HA-mHand1; the orientation was verified by *Hind*III digest. To construct the *E. coli* expression vector pGEX2T-HA-mHand1, the 699-bp fragment above was subcloned into pGEX2T at a *BamH*I site and the orientation verified by *Hind*III digest. The mHand1T107AS109A and mHand1T107DS109D mutants were obtained with a site-specific mutagenesis kit (Invitrogen) and the sequences of the two pairs of oligos were: for T107AS109A, 5′-G AAG GAG AGG AGA CGC GCA GAG GCC ATT AAC AGC GCG TTC-3′ and 5′-GAA CGC GCT GTT AAT GGC CTC TGC GCG TCT CCT CTC CTT C-3′; for T107DS109D, 5′-G AAG GAG AGG AGA CGC GAC GAG GAC ATT AAC AGC GCG TTC-3′ and 5′-GAA CGC GCT GTT AAT GTC CTC GTC GCG TCT CCT CTC CTT C-3′ (sequences for mutated amino acids are underlined). Single amino acid mutants (T107A or S109A) were acquired by a similar strategy. Mouse *Twist1* was amplified by PCR using pcDNA3-HA-Twist1 (a gift from Dr. Gerard Karsenty at the Baylor Medical College) as template and the two primers are: (forward) *BamHI* **GGA TCC** ACC (*HA tag*) *ATG GCT TAC CCA TAC GAT GTT CCA GAT TAC GCT TCG* (Twist1-5′) ATG CAG GAC GTG TCC AGC TCG; (reverse) *EcoRI* **GAA TTC** (Twist1-3′) CTA GTG GGA CGC GGA CAT. The amplified fragment was ligated into pCMV5 for mammalian cell transfection (pCMV5-HA-Twist1). The m*Twist1*T125AS127A and m*Twist1*T125DS127D mutants were obtained with a site-specific mutagenesis kit (Invitrogen) and the sequences of the two pairs of oligos were: for T125AS127A, 5′-CGG GAG CGC CAG CGC GCG CAG GCG CTG AAC GAG GCG TTC GCC GCC-3′ and 5′-GGC GGC GAA CGC CTC GTT CAG CGC CTG CGC GCG CTG GCG CTC CCG-3′; for T125DS127D, 5′-CGG GAG CGC CAG CGC GAC CAG GAC CTG AAC GAG GCG TTC GCC GCC-3′ and 5′-GGC GGC GAA CGC CTC GTT CAG GTC CTG GTC GCG CTG GCG CTC CCG-3′ (sequences for mutated amino acids are underlined). Single amino acid mutants (T127A or S127D) were acquired by a similar strategy. After mutagenesis, the sequences were verified by sequencing. pCI-E12 was obtained from Martin Knoefler (University of Vienna, Austria), the reporter plasmid of 6×Thing1-Luciferase from Paul Riley (University College London), and the ANF-luciferase reporter plasmids from Mona Nemer (University of Ottawa, Canada) and Anning Lin (University of Chicago), respectively. pCMV5-Myc-E12 was generated by PCR using pCI-E12 as a template and the product was cloned into pCMV5 at *EcoR*I and *BamH*I sites. The two primer sequence were: forward, *EcoR*I GAA TTC ACC (*Myc tag*) *ATG GAA CAA AAA CTT ATT TCT GAA GAA GAT CTG* (E12-5′) GCG CCT GTG GGC ACA GAC; reverse, *BamH*I GGA TCC (E12-3′) TCA CAT GTG CCC GGC GGG. pCMV5-myc-mTbx5 was cloned by PCR using the plasmid Tbx5-pcDNA3 from Katherine Yutzey (Cincinnati Children's Hospital Medical Center) as template.

### GST-HA-mHand1 fusion protein purification and generation of antibodies

GST-HA-mHand1 wild type, T107A, S109A, and T107AS109A fusion proteins were produced in *E. coli* with pGEX2T vectors; 0.1 mM IPTG was used to induce fusion protein production, the majority of which was in the form of inclusion bodies. To purify the fusion protein, the cell pellet was lysed by sonication in PBS containing 2 mM EDTA, 10 mM DTT, 0.5 mM PMSF and 1 mM benzamidine, followed by centrifugation. This step was repeated 2–3 times to wash the inclusion body. Purified inclusion bodies were sonicated again in PBS with 10 mM DTT, 0.1% SDS, and 10% glycerol (which helped dissolve the protein). The supernatant was collected and dialyzed overnight in PBS at 4°C. To generate a Hand1 antibody, a peptide with the sequence IPNVPADTKLSKIKTLRLATSYIA was used to immunize two rabbits after conjugation with exogenous protein (Nanjing Chuanbo Biotech. Co., Ltd.). The anti-sera were affinity purified and tested on cell transfections or mouse heart tissues by western blotting. A similar procedure was used for the generation and purification of Hand1T107pS109p phospho-specific antibody using the peptide sequence KKERRRTpESpINSAFA (Nanjing Chuanbo Biotech. Co., Ltd.).

### Cell culture, transfection, treatment and luciferase reporter assay

HEK293 cells were maintained in DMEM plus 10% fetal bovine serum (Gibco and Hyclone) and transfected using either FuGene HD (Roche) or GenEscort (Nanjing Wisegen Biotech. Co., Ltd) and plasmid amounts of 10 mg per 10-cm dish or 2 mg per well in 6-well plates. For treatment, the cells were first serum-starved for 20 h at 1 day after transfection and then treated with insulin (0.1 mM) for 15 min or LY294002 (20 mM) for 2 h prior to insulin treatment. For the luciferase reporter assay, HEK293 cells were plated into 12-well plates and transfected with 100 ng 6×Thing1 luc. plasmid or ANF-luc. plasmid, 10 ng pRL-TK-ren., 100 ng pCI-E12, 100 ng pCMV5-Hand1 wild type or mutants and 100 ng pCMV5-m/p-Akt. In other experiments, different amounts of plasmids were used, as indicated. The total amount of DNA in each treatment was normalized with pCMV5 empty plasmid. Each transfection was performed in duplicate. Cells were collected 48 h after transfection for the dual-luciferase assay using the Promega assay system (Glomax). Each assay was repeated at least three times.

### Neonatal mouse cardiomyocyte culture and lenti-viral infection

Cardiomyocytes were isolated as previously described [48]. Briefly, hearts were dissected from newborn mice and atria were removed. Ventricles were excised to pieces (1×1 mm) and incubated in collagenase (type 2, Sigma) at 37°C for 10 minutes. Subsequently, supernatants were filtered and cells in supernatants were collected by centrifugation. These cells were cultured in DMEM plus 10% fetal bovine serum (Hyclone). Most of the cells started to beat after 24 hours of culture. For lenti-viral construction, a system including lentiviral vector FuGW(ubiquitin promotor), envolope plasmid pMD_2_G and packaging plasmid pSPAX_2_ was used (a gift from Dr. Ying Wan at Department of Laboratory Animal Science, Third Military Medical University, China). Coding sequences of *Hand1*, *Twist1* and *Akt1* were obtained from pCMV5-HA-Hand1, pCMV5-HA-Twist1 and pCMV5-Flag-Akt1 and ligated into FuGW at the *BamH1* and *EcoR1* sites. Lentivirus was prepared as described in Tronolab (http://tronolab.epfl.ch). Briefly, about 4×10^6^ 293FT cells were plated in a 10 cm cell culture dish. At 80–90% confluency, the cells were co-transfected with 5 µg of envelop plasmid, 15 µg of packaging plasmid and 20 µg of lentiviral vector using calcium phosphate precipitation. After 9 h, the medium was removed and 10 ml of fresh DMEM plus10% FBS (Hyclone) was added to each dish. 48 h later, supernatant was collected and 1 ml of supernatant was added in to the neonatal mouse cardiomyocytes cultured in 6-well plate for cell infection. 24 h later, the medium was removed and 3 ml of fresh DMEM plus10% FBS (Hyclone) was added to each well. 48 h later about 80% of cells were GFP positive and cells were collected for analysis.

### *In vitro* and *in vivo* phosphorylation assay

Phosphorylation of GST-Hand1 and mutants were carried out at 30°C for 30 minutes in a volume of 50 ul of kinase assay buffer containing GST-Hand1 or mutants, hot-ATP and active Akt. The samples were subjected to SDS-PAGE followed by autoradiography. For mass spectrometric assay, bands (GST-Hand1 or HA-Hand1 from immunoprecipitation) were excised from SDS-PAGE gel for in-gel tryptic digestion and the peptides were separated by capillary liquid chromatography tandem mass spectrometry (LC-MSMS) using a Magic C18 100 µm×10 cm HPLC column (Swiss BioAnalytics, Switzerland) on a 1100 Nano-HPLC system (Agilent, Palo Alto, CA) connected on line to a 4000 Q Trap (MDS Sciex, Concord, Ontario, Canada) [49]. MASCOT searching SwissProt_57.6 was used to identify the phosphopeptides. Individual spectra were further evaluated manually.

### Western blotting analysis and immunoprecipitation

Cells and hearts were collected and snap-frozen in liquid nitrogen until use. Cell/tissue lysates were prepared in lysis buffer (20 mM Tris, 150 mM NaCl, 10% glycerol, 20 mM glycerophosphate, 1% NP40, 5 mM EDTA, 0.5 mM EGTA, 1 mM Na_3_VO_4_, 0.5 mM PMSF, 1 mM benzamidine, 1 mM DTT, 50 mM NaF, 4 mM leupeptin, at pH 8.0). Samples were resolved by 10% SDS-PAGE and transferred to PVDF membranes (Millipore). Membranes were blocked with 5% non-fat milk in TBST (50 mM Tris, 150 mM NaCl, 0.5 mM Tween-20, pH 7.5) and incubated with primary antibodies overnight at 4°C. Immunoprecipitation was performed using protein A sepharose CL-4B beads (Amersham-Pharmacia).

### Gel shift assay

Nuclear extracts from HEK293 cells overexpressing Hand1-AA and Hand1-DD were prepared using a nuclear extract kit (Active motif) according to manufacturer's instructions. Oligonucleotides containing a consensus Hand1 binding site (Th1 D-box) that wasdescribed previously were synthesized as follows: (Sense) 5′-ATTGGCGTCTGGCATTGCATTGGCGTCTGGCATTGCATTGGCGTCTGGCATT-3′; (Anti-sense) 5′–GATCTAATGCCAGACGCCAATGCAATGCCAGACGCCAATGCAATGCCAGACGCCAATGAGCT-3′ (D-box sequences were underlined) [11]. The two oligonucleotides were annealed and used as probe. Gel shift assays were performed using a gel shift assay kit (Promega) according to manufacturer's instructions.

### ChIP assay

Cell lysates and nuclear extracts were prepared from HEK293 cells over expressing HA-Hand1-WT, HA-Hand1-AA, HA-Hand1-DD and HA-m/p-AKT plus HA-Hand1-WT, respectively. The 6×Thing1 DNA fragment containing six successive Hand1 D-box (CGTCTG) sequences was prepared by *Kpn*I and *Sac*I digestion from 6× Thing1 luc vector. The nuclear extracts and 6× thing1 DNA fragment were incubated in 5× binding buffer (20% glycerol, 5 mM MgCl_2_, 2.5 mM EDTA, 2.5 mM DTT, 250 mM NaCl, 50 mM Tris-HCl (pH 7.5), 0.25 mg/ml poly(dI-dC)•poly(dI-dC)) for 30 minutes at room temperature. Immuoprecipitation was performed using HA antibody (1∶100) according to the manufacturer's instructions of Upstate ChiP assay kit. DNA was recovered by phenol/chloroform extraction and ethanol precipitation. The following PCR primers against 6× Thing1 fragment were used as follows: 5′-CATTGGCGTCTGGCATTATTG-3′ and 5′-AGCTCAATGCCAGACGCCAAT-3′.

### Mice

Mice were housed in accordance with the regulations on mouse welfare and ethics of Nanjing University in groups with 12-h dark-light cycles and free access to food and water. The experimental animal facility has been accredited by AAALAC (Association for Assessment and Accreditation of Laboratory Animal Care International) and the IACUC (Institutional Animal Care and Use Committee) of Model Animal Research Center of Nanjing University approved all animal protocols used in this study. Hand1 and Twist1 transgenic mice were generated through pro-nuclear injection of plasmid DNA. HA-Hand1, HA-Twist1 wild-type and mutants were ligated into alpha-MyHC clone 26 (a gift from Dr. Jeffrey Robbins at the Cincinnati Children's Hospital Medical Center) at *Sal*I site and the plasmids were linearized with *Not*I [50]. Founders were genotyped by PCR and expression levels were examined with western blot analysis. Progenies of founders were backcrossed to C57/B6 genetic background. Primers for genotyping of Hand1 TG mice are: forward 5′-GCTGCCTATGGTCCCGATGCCA-3′ and reverse (hGH poly A in the vector) 5′-GCACTGGGGAGGGGTCAC-3′. The PCR product was 456 bp. Primers for genotyping of Twist1 TG mice are: forward: 5′-GCGGGTCATGGCTAACG-3′ and reverse: 5′-GCACTGGGGAGGGGTCAC-3′. The size of PCR product is 400 bp.

### Echocardiography (Echo)

Echo was performed with Vevo 660 UBM (VisualSonics, Toronto, ON, Canada) that possesses a single-element mechanical transducer with a center frequency of 30 MHz and a frame rate of 30 Hz. The spatial resolution of B-mode imaging was ∼115 um (lateral) by ∼55 um (axial). Mice were anesthetized with Avertin that was prepared as a 1.2% solution and administrated to mice at a dose of 0.2 ml/10 grams body weight (approximately 240 mg/kg body weight). Body temperature of mice was monitored by rectal thermometer and was maintained between 36 and 38°C. The heart rate was maintained between 350–450 beats/min.

### Histology and immunohistochemical staining

The hematoxylin-eosin (HE) and immunohistochemical (IHC) protocols were as described previously [51], [52]. Briefly, hearts were washed with cold PBS and fixed in formalin overnight at 4°C. The samples were processed successively by (a) a 30-min wash in PBS at 4°C, (b) a 1-h incubation in 70%, 80%, 95%, and 3×1 h incubation in 100% ethanol at room temperature (RT), (c) 3×20 min incubation in xylene at RT, (d) a 1-h incubation in paraffin/xylene (1∶1) at 65°C, (e) 3×1 h incubation in fresh paraffin at 65°C. The processed samples were then embedded in paraffin and sectioned (7 µm thick) and the sections HE stained following the standard protocol. For IHC, frozen-sections were incubated at 4°C overnight with antibodies for HA and To-Pro 3 iodine (642/661, Molecular Probes). Images were processed with an Olympus confocal microscope system.

### Microarray analysis

Three hearts were dissected from each group of control, Hand1-WT, -DD and AA mice. Total RNA was isolated from each three hearts and was pooled together. In total, four RNA samples were applied for microarray analysis (Affymetrix Mouse 430 2.0 Array, Shanghai Biochip Co. Ltd.). The data was analyzed by use of Molecular Annotation System (Beijing CapitalBio Corporation).

### Masson's trichrome staining

Myocardial fibrosis in myocardial infarct mice were detected by masson's trichrome staining. Hearts were cross-sectioned in 6 µm thick and then stained accroding to the protocol (http://www.ihcworld.com/_protocols/special_stains/masson_trichrome.htm).

### Statistics

Data were presented as mean±SD values. For comparisons between two groups, statistical significance was determined using the unpaired two-tailed *t*-test. A value of *P*<0.05(*) was considered statistically significant and *P*<0.01(**) statistically very significant.

## Supporting Information

Figure S1 **A. Analysis of gene expression in Hand1 TG hearts by RT-PCR.** Three individual hearts for each group were analyzed. * indicates up-regulation in –AA and –DD hearts compared to control and –WT. **B**. Quantitation of **A**, * represents *P*<0.05. **C**. RT-PCR analysis of total Hand1 expression in multiple tissues of TG mice.
(TIF)

Figure S2 **Study of Twist1 phosphorylation by Akt in U2OS cells.**
(TIF)

Table S1 **Microarray analysis of Hand1 TG hearts**
(DOC)
