# Supplementary figures and images for: Phosphorylation of the Twist1-Family Basic Helix-Loop-Helix Transcription Factors Is Involved in Pathological Cardiac Remodeling
Source: PLoS One. 2011 Apr 29;6(4):e19251. doi: 10.1371/journal.pone.0019251 (PMC3084786; doi:10.1371/journal.pone.0019251)

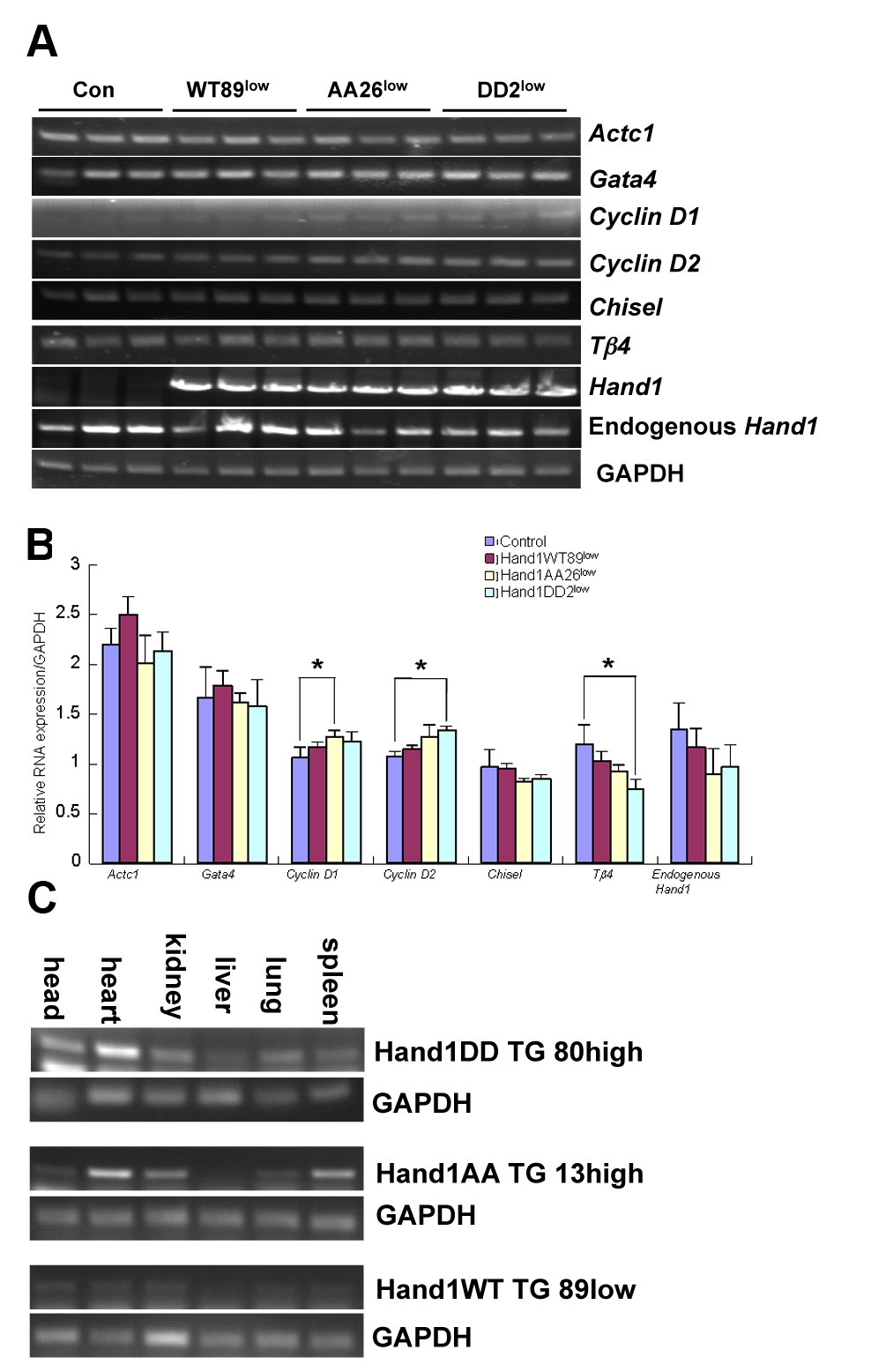

Supplement: Figure S1 — A. Analysis of gene expression in Hand1 TG hearts by RT-PCR. Three individual hearts for each group were analyzed. * indicates up-regulation in –AA and –DD hearts compared to control and –WT. B. Quantitation of A, * represents P<0.05. C. RT-PCR analysis of total Hand1 expression in multiple tissues of TG mice. (TIF) [file pone.0019251.s001.tif]

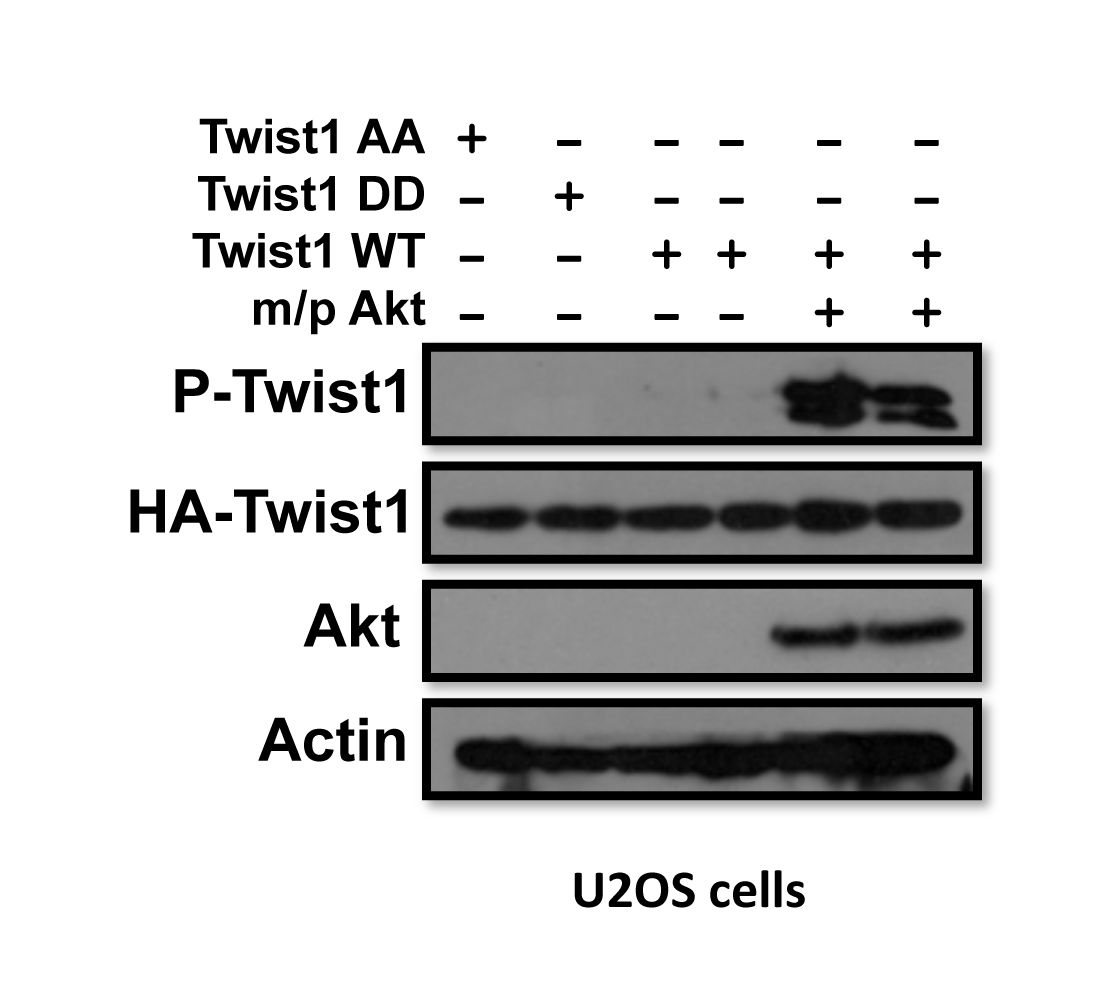

Supplement: Figure S2 — Study of Twist1 phosphorylation by Akt in U2OS cells. (TIF) [file pone.0019251.s002.tif]
