# Supplementary material for: Phosphorylation of the Twist1-Family Basic Helix-Loop-Helix Transcription Factors Is Involved in Pathological Cardiac Remodeling
Source: PLoS One. 2011 Apr 29;6(4):e19251. doi: 10.1371/journal.pone.0019251 (PMC3084786; doi:10.1371/journal.pone.0019251)
Supplement: Table S1 — Microarray analysis of Hand1 TG hearts (DOC) [file pone.0019251.s003.doc]

**Table S1**. Microarray analysis of Hand1 TG hearts

| **Hand1 DD vs WT** | | |
| --- | --- | --- |
| **Pathway** | **Gene symbol** | **Fold change** |
| **Regulation of actin cytoskeleton** | Itgav | ↑2.8284 |
|  | Fgfr1 | ↑2.2974 |
|  | Iqgap1 | ↑2.4623 |
|  | F2r | ↑3.4822 |
|  | Abi2 | ↑3.0314 |
|  | Actn4 | ↑2.1435 |
|  | Fgf12 | ↑3.4822 |
|  | Ssh1 | ↑2.2974 |
|  | Gna13 | ↑2.2974 |
|  | Itga8 | ↑2.6390 |
|  | Arpc2 | ↑2.8284 |
|  | Actn1 | ↑2.4623 |
| **Focal adhesion,**  **ECM-receptor interaction** | Col3a1 | ↑5.2780 |
|  | Igf1r | ↑3.4822 |
|  | Col5a2 | ↑5.6569 |
|  | Igf1 | ↑4.5948 |
|  | Col1a2 | ↑6.4980 |
|  | Zyx | ↑2.1435 |
|  | Parva | ↑2.4623 |
|  | Ccnd2 (cyclin D2) | ↑4.9246 |
|  | Col6a2 | ↑2.4622 |
|  | cd44 | ↑2.4623 |
|  | Sdc4 | ↑4.2871 |
| **Tight junction** | Gnai2 | ↑2 |
|  | Epb4.1 | ↑2 |
|  | Cldn5 | ↑4.2871 |
|  | Ppp2ca | ↑2 |
|  | Myh11 | ↑2 |
|  | Spnb2 | ↑2.6390 |
|  | Myh7 | ↑14.9285 |
| **Oxidative phosphorylation** | Ndufa4 | ↓2 |
|  | Atp5g2 | ↓2 |
|  | Ndufab1 | ↓2.1435 |
|  | Atp5a1 | ↓2 |
|  | Ndufa9 | ↓2 |
|  | Uqcrc2 | ↓2 |
|  | Ndufs8 | ↓2.1435 |
|  | Atp6v1c1 | ↑2.1435 |
|  | Cox7a1 | ↓2.6390 |
|  | Sdha | ↓2.8284 |
|  | Atp5j | ↓2 |
|  | Atp5c1 | ↓2 |
|  | Ndufv2 | ↓2.1435 |
|  | Sdhd | ↓2.8284 |
| **Citrate cycle (TCA cycle)** | Idh2 | ↓2.2974 |
|  | Dld | ↓4 |
|  | Cs | ↓4.2871 |
|  | Sucla2 | ↓2.1435 |
|  | Idh3a | ↓4 |
|  | Mdh1 | ↓2 |

| **Hand1 AA vs WT** | | |
| --- | --- | --- |
| **Pathway** | **Gene symbol** | **Fold change** |
| **Focal adhesion, ECM-receptor interaction, Cell Communication** | Ccnd2 (CyclinD2) | ↑2 |
|  | Col1a2 | ↑2.1435 |
|  | Ccnd1 (CyclinD1) | ↑2.2974 |
|  | Col1a1 | ↑2.4623 |
|  | Col5a2 | ↑2.6390 |
|  | Col3a1 | ↑3.7321 |
|  | Thbs4 | ↑7.4643 |
